# Characteristics and Spatial–Temporal Differences of Urban “Production, Living and Ecological” Environmental Quality in China

**DOI:** 10.3390/ijerph192215320

**Published:** 2022-11-19

**Authors:** Le Zhang, Qinyi Gu, Chen Li, Yi Huang

**Affiliations:** 1 School of Marxism, Jiangnan University, Wuxi 214122, China; 2 School of Management, Shanghai University of Engineering Science, Shanghai 201620, China; 3 School of Geographic Sciences, Nantong University, Nantong 226019, China

**Keywords:** urban “production, living and ecology” environmental quality, spatial and temporal differences, entropy method, Chinese cities

## Abstract

The article analyses the spatial and temporal differences in the environmental quality of production, living and ecology of 285 cities in China from 2010 to 2020 by using the entropy method, the Theil index and correlation analysis. The study concludes the following: (1) in terms of overall differences, the overall differences in the “production, living and ecological” environmental quality indices of 285 cities during the study period undergo a process of “narrowing–widening–narrowing”. The differences within the four major zones of the country are higher than those between the four major zones, and the differences within the zones show an increasing trend year by year. (2) In terms of temporal differences, the combined scores of “production, living and ecological” environmental quality of the 285 cities in the study period show a decreasing trend, and the contribution of the PLE subsystem scores are, in descending order, production environmental quality > living environmental quality > ecological environmental quality. (3) In terms of overall ranking, the head effect of the combined production, living and ecological environmental quality (PLE) scores of cities in the study period is significant, and the top 10 cities in terms of combined scores are all small and medium-sized cities with significant regionalization characteristics. (4) In terms of spatial pattern, there is a significant spatial gradient in the east, central and western regions, with the overall PLE scores of the four major regions in descending order: eastern region > central region > western region > northeastern region. The regions with high scores in the “production, living and ecological” environmental quality of cities can be divided into three types: multi-core, dual-core and single-core. (5) In terms of influencing factors, there is a logarithmic curve relationship between the combined production, living and ecological environmental quality (PLE) score and the built-up area (BUA) of cities. The study proposes to optimize the layout of urban production, strengthen the industrial links of urban clusters, improve the level of public services, ensure the equalization of urban public services, strengthen the management of ecological environment and improve the quality of ecological environment in order to optimize the quality of urban “production, living and ecological” environment.

## 1. Introduction

With rapid industrialization and urbanization, China’s economy and society have grown at a rapid pace, with the gross regional product rising from US $149.5 billion in 1978 to US $17.73 trillion in 2021, and the urbanization rate rising from 17.90% in 1978 to 64.72% in 2021. However, the rapid urbanization development has brought about increasingly serious problems. China’s development is facing the crisis and challenge of approaching resource and environmental constraints, increasing environmental pollution and degradation of ecosystems [1]. The quadrennial report of the UN Secretary-General on progress in the implementation of the New Urban Agenda states that “rapid urbanization, climate threats to our ecosystem and the profound impact of the global COVID-19 pandemic are among the top challenges faced by the world today. These problems create daily stress for our cities and human settlements. At the same time, cities provide opportunities to anchor the pandemic recovery in social justice, deliver the 2030 Global Agenda commitments and achieve national climate targets under the Paris Agreement. If managed and planned sustainably and equitably, cities offer solutions to address social and environmental issues” [2]. From the perspective of habitat science [3,4,5], the enormous pressures on cities and human settlements are essentially the result of an imbalance in the quality of the “production, living and ecological” environment, the result of years of uncontrolled use and over-exploitation. This shows that the crude form of economic growth is not sustainable.

In order to change the mode of economic growth, we must follow the path of sustainable development, respect the laws of urban development, implement the new development concept of innovation, coordination, green, openness and sharing, meet the growing needs of the people for a better life, and support the role of the United Nations development system in implementing sustainable development goals. Western developed countries have all faced the same dilemma in the process of industrialization, and they have all adopted development strategies that optimize territorial space and attach importance to environmental protection [6,7,8,9,10]. These experiences are of great significance in guiding China to solve the current dilemma of sustainable development. The evaluation system of “production, living and ecological” environmental quality in cities is the basis for the balanced development of “production, living and ecological” environment. A large number of studies have been conducted and many important research results have been achieved, including a series of studies on the quality of production environment and its influencing factors [11,12,13,14,15,16,17,18], a series of studies on the quality of life and its influencing factors [19,20,21,22,23,24,25,26,27], and a series of studies on water, soil and gas pollution and related environmental pollution [28,29,30,31,32,33,34,35,36,37].

Firstly, researchers have analyzed different perspectives on the integrated assessment of the “production, living and ecological” environmental quality of cities. ① Research on indicators for the spatial allocation of “production, living and ecology” resources. The scientific issues of the quantity ratio and spatial allocation of “production, living and ecological” space was elaborated [38], and it was pointed out that the theory of “production, living and ecological space” is a new form derived from the combination of the theory of “social–economic–natural composite ecosystem” and the practice of urban and rural planning [39]. ② Research on the indicators of the spatial function of “production, life and ecology” resources. Research on the main functions of “production, living and ecology” land [40,41], construction of a classification system of “production, living and ecological land” [42], establishment of a comprehensive evaluation index of the quality of land use and its subsystem quality of production, living and ecological space use [43], and establishment of an index of the quality of land use to quantitatively measure the functional value of production, living and ecological space [44] were proposed. ③ Research on the indicators of “production, living and ecological” resources and spatial land use. The spatial scale variability, spatial function compounding, spatial scope dynamics and spatial land use heterogeneity of the objects under the concept of “production, living and ecological space” were analyzed [45], and a production, living and ecological space classification and evaluation system was established [46]. ④ Research on the indicators of the carrying capacity of the “production, living and ecology” system. The ecological, production and living carrying capacity of land is made up of the ecological, production and living carrying capacity of land [47]. ⑤ Research on the indicators of the agricultural system of “production, life and ecology”. From the perspective of spatial reconstruction of production, life and ecology, the spatial optimization of the layout of rural settlements was carried out [48].

Secondly, the evaluation of the quality of the urban “production, living and ecology” environment was performed. ① The spatial perspective of “production, life and ecology”. Based on the perspective of “production, living and ecological space”, a spatial evaluation system of production, living and ecological functions was constructed [49]. ② The perspective of net initial productivity. Using the theories of efficiency, quality and integration in the spatial use of national land, and the ecological spatial assessment model of net primary productivity (NPP), a spatial zoning index system for production, living and ecology was constructed [50]. ③ The perspective of national census. According to the Content and Indicators of the National Geographic Census, the production, living and ecological spaces are divided into production space, production and ecological space, living space, living and production space, ecological space, ecological production space and ecological living space [51]. ④ A systems theory perspective. Under the perspective of system theory, the ecology, production and life of wetlands are integrated to construct a wetland ecology–economy–society composite system, and the system dynamic characteristics of causal characteristics, multiple feedback characteristics, system nonlinearity and system inertia that exist in this composite system are analyzed [52]. ⑤ Perspective of national land classification. With reference to the national land use classification method combined with the LUCC classification system, a production, living and ecological spatial classification and evaluation system based on the principle of multi-functionality of land use was constructed on the basis of the land use function [53].

Thirdly, the analysis of the factors influencing the quality of the urban “production, living and ecological” environment was conducted. ① The study of the factors influencing production space. To measure the spatial and temporal efficiency of industrial production space, the spatial and temporal evolution characteristics and driving mechanisms of the industrial production space in the Pearl River Delta city cluster were revealed [54], the spatial pattern and influencing factors of industrial production efficiency in Jiangsu were measured using the stochastic frontier production function [55], and the regional differences in green production efficiency in China were measured [56]. ② Research on the influencing factors of living space. The study investigated the influence of housing factors on the happiness of urban residents [57], and measured the satisfaction of residents’ life by using multi-level fixed-order dependent variables [58]. ③ Research on the influence of ecological space. Research on the evaluation and influencing factors of ecological environment and ecological space, such as the evaluation of “beautiful China” by ecological status theory [59], was conducted.

In summary, the above-mentioned studies provide a solid analytical basis for this paper, as scholars have discussed the issue of urban “production, living and ecological” environmental quality, but have focused more on the spatial land use aspect of “production, living and ecological”, lacking a comprehensive measurement of urban “production, living and ecological” environmental quality, and lacking spatial and temporal analysis of continuous data. This paper is based on the data matrix of the “production, living and ecological” environmental quality of 285 cities in China from 2010 to 2020, and reveals the spatial and temporal characteristics of the “production, living and ecological” environmental quality of cities. This paper will be divided into four parts. The first part is the introduction, which introduces the progress of research on urban “production, living and ecological” environmental quality; the second part is the methods and data, which introduces the research methods, indicator system and data sources; the third part is the results and analysis, which is the main focus of the paper, and the research will develop the empirical analysis of the overall differences, time-series differences, comprehensive rankings, spatial and temporal patterns of the “production, living and ecological” environmental quality of cities and their influencing factors; the fourth part is the main conclusion of the paper, and proposes targeted countermeasures.

## 2. Methods and Data

Our study covers 285 cities at prefecture level and above in China, which are located in the eastern, central, western and northeastern regions of China, with 86, 80, 85 and 34 cities involved in the four major regions, respectively. The study includes all important cities in China, comprising municipalities directly under the central government, provincial capitals and large cities, and the samples are representative and significant. Our study will measure the “production, living and ecological” environmental quality of 285 cities and analyze their subsystems to reveal the overall differences, time-series differences, overall rankings and spatial and temporal patterns in the development of the “production, living and ecological” environmental quality of cities. 

### 2.1. Methods

#### 2.1.1. Entropy Method

This study uses the entropy method to comprehensively evaluate the level of PLE of 285 prefecture-level and above cities in China. The entropy method can profoundly reflect the utility value of the entropy value of indicator information, and the indicator weight value given has higher credibility than the hierarchical analysis method and the expert experience assessment method, which is suitable for the comprehensive evaluation of multiple indicators, and its main steps are [60] described below.

Step one: construct the original indicator data matrix. Assume there are *m* programs to be evaluated and *n* evaluation indicators, forming the original indicator data matrix:

(1) $$X={\{x_{i}{}_{j}\}}_{m\times n}(0\leq i\leq m,0\leq j\leq n)$$

where *x_ij_* is the indicator value of the *j*th indicator of the *i*th program to be evaluated.

Step two: data standardization. As the scale, order of magnitude and positive and negative orientation of the indicators are different, the initial data should be standardized.

(2) $${x^{\prime }}_{ij}=\left\{ \begin{array}{l} \frac{x_{ij}-x_{ij\min}}{x_{ij\max}-x_{ij\min}}\\ \frac{x_{ij\max}-x_{ij}}{x_{ij\max}-x_{ij\min}} \end{array}\right.$$

Define the normalization matrix:

(3) $$y_{ij}=\frac{{x^{\prime }}_{ij}}{\sum {x^{\prime }}_{ij}},0\leq y_{ij}\leq 1$$

Step three: let *k* = 1/ln*m*, and calculate the entropy value of the evaluation indicator:

(4) $$e_{j}=-k\sum (y_{ij}\times \ln y_{ij})=(\frac{1}{\ln m})\sum \left(y_{ij}\times \ln y_{ij}\right)$$

Step four: calculate the coefficient of variability of the evaluation indicators and define the weights of the evaluation indicators.

(5) $$g_{j}=1-e_{j}$$

(6) $$w_{j}=g_{j}/\Sigma g_{j}$$

Step five: calculate the evaluation value of the sample. The product of the weight *w_j_* of the *j*th indicator and the proximity *x′_ij_* of the *j*th evaluation indicator of the *i*th sample in the standardized matrix is used as the evaluation value *f_ij_* of *x_ij_*, and the evaluation value *f_i_* of the *i*th sample.

(7) $$f_{ij}=w_{j}\times {x^{\prime }}_{ij}$$

(8) $$f_{i}=\Sigma f_{ij}$$

#### 2.1.2. Theil Index

Theil index examines inequality and disparity from the concepts of information quantity and entropy, and it decomposes overall disparity into disparity between parts and disparity within parts, which has wide applications for analyzing and decomposing disparity [61,62,63]. The composite entropy index examines the variability among individuals from the concepts of information quantity and entropy, which is the expected value of information quantity, i.e., the expected information quantity [64,65]. The closer the individuals are to each other, the smaller the composite entropy index will be.

(9) $$GE=\left\{ \begin{array}{l} \sum_{i=1}^{n}p_{i}[{\left(y_{i}/u\right)}^{c}-1],c\neq 0,1\\ \sum_{i=1}^{n}p_{i}(y_{i}/u)lg(y_{i}/u),c=1\\ \sum_{i=1}^{n}p_{i}lg(y_{i}/u),c=0 \end{array}\right.$$

In Equation (9), the parameter c is used to determine the sensitivity of the exponential change. In general, when c < 2, the exponential change it determines is sensitive. When c = 0.1, it is the well-known Theil index.

Due to its property of dividing overall differences into within-group differences and between-group differences, the Theil index is widely used in empirical studies of overall spatial heterogeneity, as well as inter-spatial heterogeneity. The calculation formula is

(10) $$\mathrm{Theil}=\sum_{i=1}^{n}T_{i}ln(nT_{i})=T_{WR}+T_{BR}$$

If the area under study is divided into groups according to certain methods, the Theil index can be further decomposed into intra-group differences and inter-group differences.

(11) $$T_{WR}=\sum_{i=1}^{n_{db}}T_{i}ln(n_{db}\frac{T_{i}}{T_{db}})+\sum_{i=1}^{n_{d}}T_{i}ln(n_{d}\frac{T_{i}}{T_{d}})+\sum_{i=1}^{n_{z}}T_{i}ln(n_{z}\frac{T_{i}}{T_{z}})+\sum_{i=1}^{n_{x}}T_{i}ln(n_{x}\frac{T_{i}}{T_{x}}),$$

(12) $$T_{BR}=T_{db}ln(T_{db}\frac{n}{n_{db}})+T_{d}ln(T_{d}\frac{n}{n_{d}})+T_{z}ln(T_{z}\frac{n}{n_{z}})+T_{x}ln(T_{x}\frac{n}{n_{x}}),$$

In Equations (10)–(12), Theil is Theil index; n is the number of cities in the sample region; T_WR_ is the differences within the four regional groups of northeast, east, central, and west regions; T_BR_ is the differences between the four regional groups; n_db_, n_d_, n_z_, and n_x_ are the number of cities within the northeast, eastern, central, and western regions, respectively; T_i_ is the carbon emission index of region in and the national average ratio; T_db_, T_d_, T_z_, and Tx are the ratios of carbon emission indexes of the northeast, east, central, and west regions to the national average, respectively. A total of 285 cities at the prefecture level and above are included in our study. The northeast region includes 34 cities in Liaoning, Jilin, and Heilongjiang; the east region includes 86 cities in Beijing, Tianjin, Hebei, Shanghai, Jiangsu, Zhejiang, Shandong, Fujian, Guangdong, and Hainan; the central region includes 80 cities in Shanxi, Henan, Anhui, Hubei, Hunan, and Jiangxi, and the west region includes 85 cities in Inner Mongolia, Chongqing, Sichuan, Guangxi, Guizhou, Yunnan, Shaanxi, Gansu, Ningxia, Tibet, Qinghai, and Xinjiang.

#### 2.1.3. Correlation Analysis

The scatter plot is the most visual method used to express correlation analysis. The correlation coefficient is a collective term for a class of indicators that measure the correlation between variables [66,67,68].

The common correlations are linear correlation, curvilinear correlation, positive correlation, and negative correlation. The Pearson correlation coefficient, also known as the product–difference correlation coefficient, is a common metric for quantitatively describing the degree of linear correlation [69,70,71,72]. The formula for measuring the Pearson correlation coefficient is the following:

(13) $$r=\frac{\sum \left(x_{i}-\overset{-}{x})(y_{i}-\overset{-}{y}\right)}{\sqrt{{\left(x_{i}-\overset{-}{x}\right)}^{2}}\sqrt{{\left(y_{i}-\overset{-}{y}\right)}^{2}}}$$

In Equation (13), x_i_ and y_i_ are the variables, $\overset{-}{x}$ and $\overset{-}{y}$ are the means of variables x_i_ and y_i_, and r is the correlation coefficient. The maximum correlation coefficient is 1. The closer the absolute value of the correlation coefficient is to 1, the stronger the correlation between the variables. Our study will measure the correlation coefficient between PLE and BUA using correlation coefficients, and visually express the relationship between them by means of scatter plots.

### 2.2. Evaluation Indicators

The index system for the evaluation of the “production, living and ecological” environmental quality of the city consists of a target level, secondary indicators and tertiary indicators. The target level includes three aspects: production environment quality, living environment quality and ecological environment quality. The secondary indicators of production environment quality cover four aspects: industry, wholesale and retail, postal and telecommunications, and energy use. The secondary indicators for the quality of the living environment cover four aspects: employment, education, healthcare and social security. The secondary indicators for the quality of the ecological environment cover three aspects: greening, industrial waste discharge and environmental management. There are a total of 24 specific evaluation indicators for the three levels of indicators, of which 21 have positive attributes and 3 have negative attributes (Table 1).

### 2.3. Data Sources

The indicator data involved in our study were collected from secondary data publicly available to the government and processed appropriately. Data on the quality of the urban “production, living and ecological” environment are mainly obtained from the 2011–2021 China Urban Statistical Yearbook compiled by the National Bureau of Statistics of China and the China Urban Construction Statistical Yearbook for the past years. Data on the population size of urban areas were obtained from the “2020 China Population Census Sub-County Information” published in October 2022 and compiled by the State Council’s Seventh National Census Leading Group Office.

## 3. Analysis of the Results

### 3.1. Significant Differences in the Overall Quality of the Urban “Production, Living and Ecological” Environment

The overall difference in the “production, living and ecological” environmental quality scores of 285 cities in China between 2010 and 2020 underwent a process of “narrowing–widening–narrowing”. The Theil Index narrowed from 0.490 in 2010 to 0.466 in 2012, then widened to 0.543 in 2016, and then narrowed to 0.488 in 2020 (Table 2). Although the overall difference between the combined “production, living and ecological” environmental quality scores in 2010 and 2020 is not significant, the internal structure of the Theil Index has changed significantly.

**Figure 1:**
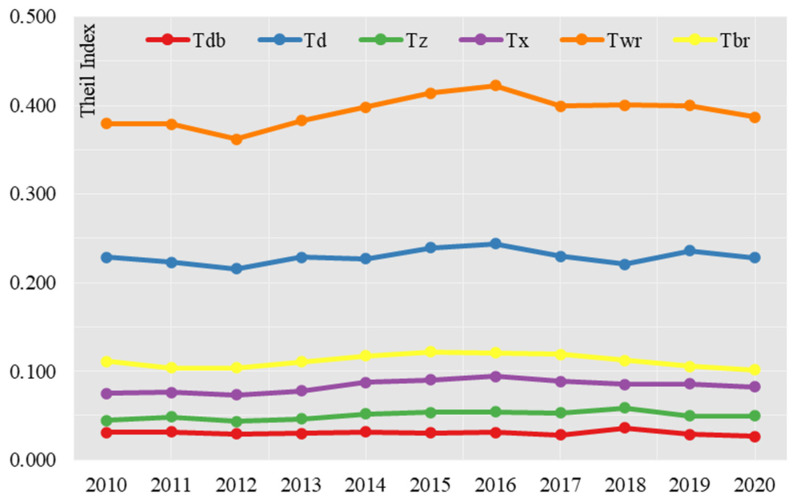
Decomposition of regional differences in urban “production, living and ecological” environmental quality scores for the period 2010–2020.

In terms of trends, the differences between the northeast, east, central and west zones show a general trend of “narrowing–expanding–narrowing”, and the differences in the environmental quality indices of “production, living and ecology” between the four zones also show a trend of “narrowing–expanding–narrowing” (Figure 1). The differences in environmental quality indices of production, life and ecology between the four zones also show a development trend of “narrowing–expanding–narrowing” (Figure 1). From the decomposition of intra-belt differences, the differences in the environmental quality indices of production, living and ecology in the Northeast region did not change much in the rest of the years, except for 2018, when the differences within the zones widened. In the eastern region, the intra-zone variation in the production, living and ecology environmental quality index fluctuated more, with the Theil index showing a fluctuating process of “narrowing–widening–narrowing–widening–narrowing”. The situation in the Central Region is similar to that in the Northeast Region, with the exception of 2018, when the internal variation widened, but the variation in the rest of the years was not significant. In the Western region, the variance index within the belt shows a “widening–shrinking” trend, but with a smaller change.

In terms of contribution rate, the differences within the four major zones of the country are much higher than the differences between the four major zones, and the differences within the zones show an increasing trend year by year (Table 3). The contribution rate of intra-belt differences increased from 77.34% in 2010 to 79.20% in 2020, while the contribution rate of inter-belt differences showed a decreasing trend year by year, decreasing from 22.66% in 2010 to 20.80% in 2020. The intra-belt contribution rate is, in descending order, Eastern Region > Western Region > Central Region > Northeast Region. The contribution rate of internal differences in the eastern region fluctuated between 43.06% and 46.73%, the contribution rate of internal differences in the western region increased from 15.32% in 2010 to 16.89% in 2020, the contribution rate of internal differences in the central region increased from 9.07% to 10.20%, and the contribution rate of internal differences in the northeastern region decreased from 6.27% to 5.39%.

In summary, internal differences in the four major zones were the main drivers of variation in the quality of the urban “production, living and ecological” environment, with internal differences in the eastern region being the main driver, while internal differences in the north-eastern region contributed the least, and internal differences in the central and western regions were second only to those in the eastern region.

### 3.2. Significant Differences in the Temporal Sequence of “Production, Living and Ecological” Environmental Quality in Cities

**Figure 2:**
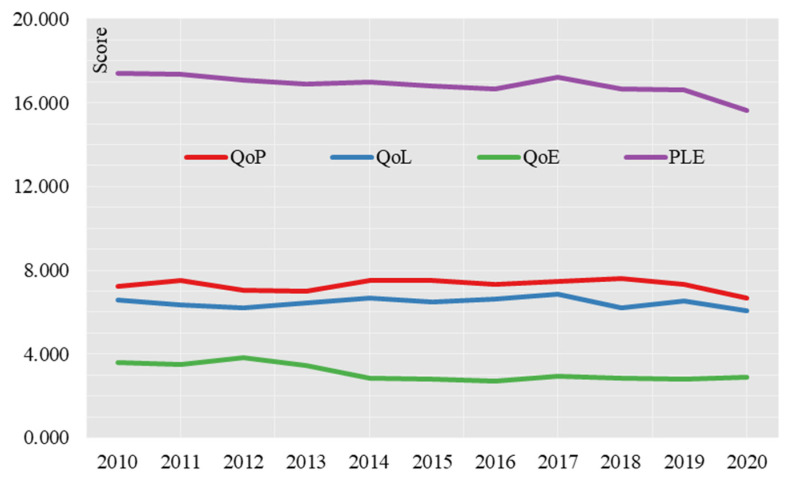
Time series changes in the city’s “production, living and ecological” environmental quality scores, 2010–2020.

The PLE scores of the 285 cities were summed to obtain the changes in the PLE scores from 2010 to 2020 (Figure 2). The overall PLE score shows a decreasing trend, from 17.386 in 2010 to 15.630 in 2020. The production environment quality score shrinks from 7.246 in 2010 to 6.676 in 2020, the living environment quality score shrinks from 6.572 in 2010 to 6.084 in 2020, and the ecological environment quality score shrinks from 3.568 in 2010 to 2.870 in 2020. The decrease in the “production, living and ecological” environmental quality scores correlate with the overall decrease in its regional difference index, reflecting the unsatisfactory level of the overall “production, living and ecological” environmental quality across the country, and the uneven development between the production, living and ecological environments. The unbalanced development between the production environment, the living environment and the ecological environment is still a prominent problem.

In terms of the score contribution of production environment, living environment and ecological environment (Table 4), the score contribution of quality of production environment (QoP) is the largest and has an overall increasing trend, with the contribution of QoP index increasing from 41.68% in 2010 to 42.71% in 2020, while the score contribution of quality of ecological environment (QoE) is the smallest and has an overall decreasing trend, with the contribution of QoE index increasing from 20.52% in 2010 to 18.36% in 2020. Quality of Living Environment (QoL) scores are in the middle of the range, with the contribution of the QoL index increasing from 37.80% in 2010 to 38.92% in 2020.

Analysis of factors influencing changes in production environment, living environment, and ecological scores was performed. Firstly, data structural factors were considered. As the differences of some indicators are relatively small, the entropy method of processing brought about changes in weights, resulting in a low score for this part of the indicators, while the differences of some indicators are large and the weights given are high, resulting in a relatively high score for this part of the indicators. Secondly, the deep transformation of China’s economic structure was considered. The study uses the number of large-scale industrial enterprises and the number of foreign-invested enterprises as positive indicators in consideration of its importance to the national economy and social development. Currently, one of China’s greatest strengths is its manufacturing sector, and to hold on to it and to deal with issues such as supply-side shocks in the face of exposure to the risks of counter-globalization, there is an urgent need to open up all aspects of production, distribution, circulation and consumption, and to promote a freer flow of production factors. Relatively speaking, the significant reduction in emissions of the “three wastes” by Chinese enterprises indicates that the government and enterprises have been effective in managing the ecological environment; industrial wastewater, industrial Sulphur dioxide and industrial smoke (dust) emissions in 285 cities were reduced from 225,493,900 tonnes, 16,978,337 tonnes and 54,165,563 tonnes in 2010 to 12,671,570 tonnes, 229,770 tonnes and 4,221,750 tonnes in 2020, a reduction of 43.81%, 86.47% and 22.06%, respectively. At the same time, China’s economic structure is undergoing a deep transformation, the energy structure, economic structure and industrial structure are being adjusted at an accelerated pace, and the manufacturing industry has started to move from the middle and low end to the middle and high end. The number of industrial enterprises above the national scale decreased from 452,872 in 2010 to 399,375 in 2020, a decrease of 11.81%, while the total profit of industrial enterprises above the national scale rose from 530.5 billion yuan in 2010 to 68.465 billion yuan in 2020, a rise of 29.06%, and the number of industrial enterprises above the scale indicator of the “one down, one up” indicates that its intrinsic structure is undergoing deep adjustments and the trend of industrial scale and concentration is strengthening. Thirdly, the impact of major public health events was considered. In response to COVID-19, China has adopted a strict epidemic prevention policy, adopting a general policy of “dynamic zero” means to eliminate an outbreak if one is detected so that there is no continuous community transmission or large-scale rebound. When a city is sealed, the primary impact is on the industry. The data is visualized in the fact that the scores for both Quality of Production Environment (QoP) and Quality of Living Environment (QoL) drop from 7.308 and 6.552 in 2019 to 6.676 and 6.084 in 2020, a drop of 8.65% and 7.14%, respectively. For example, Shanghai was sealed in March–June 2022. As a result, people found that Shanghai has a very important position in China, and the sealing of Shanghai directly impacted China’s secondary industry. Shanghai is not only an international shipping center and financial center, but also the economic center of China. The world’s top industries are in Shanghai, and the Yangtze River Delta city cluster, the engine of China’s economic growth, was directly affected after Shanghai was sealed, and after the lifting of the sealing China’s economy has only gradually recovered.

### 3.3. The Head Effect of the City’s “Production, Living and Ecological” Environmental Quality Is Significant

The production, living and ecological environmental quality (PLE) of cities shows a horse-trading effect, with the top scores being those of mega-cities or large cities, and the bottom scores being those of small and medium-sized cities (Table 5).

The top 10 cities in terms of PLE score are all mega-cities or large cities in Chin. From 2010 to 2020, Shanghai, Beijing, Guangzhou and Shenzhen, the most economically developed mega-cities in China, have maintained their PLE scores in the top four, with Shanghai consistently ranking first. Cities such as Shanghai, Beijing, Guangzhou, Shenzhen, Suzhou, Chongqing, Chengdu, Hangzhou, Dongguan and Tianjin are pivotal and important in China’s economic growth. These cities have large populations, concentrated production, and maintain full national rankings in terms of ecological and environmental quality and public service levels. Due to the concentration of production, job opportunities, development opportunities and ecological livability, the head cities pose a strong attraction to migrant populations and produce a strong siphoning effect on factors of production such as human, capital and technological resources, and are the growth poles of regional economic development. With the exception of Chengdu and Chongqing in the western region, the top cities with the highest PLE scores are mainly located in the eastern coastal region, because the eastern coastal region has excellent location conditions and is actively participating in the global division of labor, playing a leading role in linking Chinese industries to the world industrial system.

The bottom 10 cities in terms of PLE score are all small and medium-sized cities with significant regional characteristics. In 2010, the bottom 10 cities in terms of PLE are mainly located in the western region, namely Fangchenggang, Longnan, Zhangye, Chongzuo, Qitaihe, Pingliang, Lincang, Zhongwei, Zhangjiajie and Baoshan. The bottom 10 cities in terms of “production, living and ecological” environmental quality (PLE) in 2020 are mainly located in the western region and the northeastern region: Hegang, Heihe, Shuangyashan, Liaoyuan, Pu’er, Tongchuan, Jinchang, Zhongwei, Fangchenggang and Wuzhong. In terms of spatial and temporal characteristics, the bottom 10 cities in the PLE composite score from 2010 to 2020 gradually change from a westernized character to a westernized and northeasterlies character. We performed an analysis of the reasons for the bottom 10 cities in the composite score. Firstly, some cities in the western region have a thin industrial base and public service level bottom, and the ecological and environmental quality in the process of rough economic growth is not good, which together lead to some cities’ low composite PLE score. Secondly, as the state attaches importance to ecological and environmental management, the degree of investment has deepened, prompting the overall ecological and environmental quality in the western region to rise, causing some cities in the western region to gradually move out of the bottom 10. Thirdly, in recent years, the hollowing out of industries, the loss of population and the decline in the level of public services in some cities in the Northeast have led to a significant reduction in the quality of the production, living and ecological environment (PLE) in the region. For example, in Hegang, one of the most popular cities on the internet, individual houses are sold for only RMB 15,000, which is equivalent to a month’s salary in Shanghai. In an era of high property prices, Hegang has become a place for young people to escape the fierce competitive large cities. However, the issue of strengthening the resilience of industrial and supply chains, revitalizing existing resources, building a modern economic system and improving the quality of the “production, living and ecological” environment (PLE) in some northeastern cities with “hollowed out” industries and high population outflows remains a huge challenge for local governments.

### 3.4. Cities with High “Production, Living and Ecological” Environmental Quality Are Distributed in “Clusters”

The spatial gradient between the eastern, central and western regions is significant in terms of the overall scores of the four regions (Table 6). The overall scores of “production, living and ecological” environmental quality of cities in the four regions are in descending order: Eastern region > Central region > Western region > Northeast region. In 2010, the number of cities in the Top 25% Eastern, Central, Western and Northeastern regions were 47, 8, 10 and 6, respectively, and in 2020 they will be 45, 11, 11 and 4, respectively. The number of cities in the Eastern, Central, Western and Northeastern regions with a combined score of middle 50% in 2010 is 37, 62, 25 and 18, respectively, becoming 38, 57, 37 and 10, respectively, in 2020. In 2010, the number of cities in the eastern, central, western and north-eastern regions at the bottom 25% of the overall score will be 2, 10, 50 and 10, respectively, and in 2020 they will be 3, 12, 37 and 20, respectively. The biggest changes in the ranking of the four regions in terms of the “production, living and ecological” environmental quality are in the western region and the northeastern region, with some cities in the former showing a significant upward trend in the ranking, and the latter showing a significant downward trend in the ranking.

**Figure 3:**
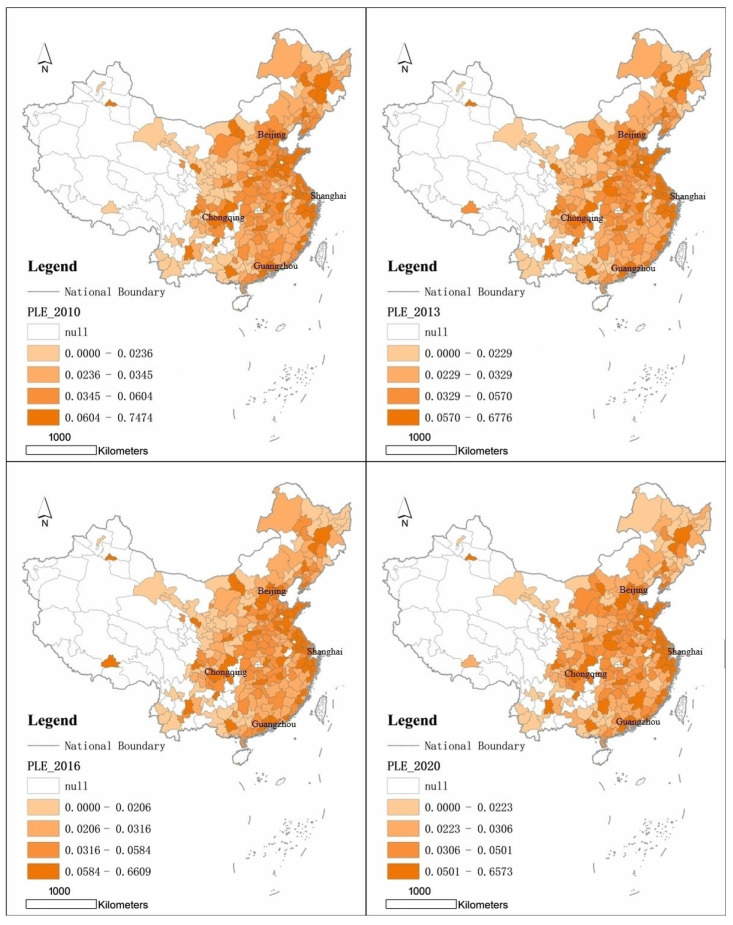
Spatial and temporal patterns of “production, living and ecological” environmental quality scores in cities, 2010–2020.

The spatial and temporal distribution of the 285 cities in terms of their overall scores shows “cluster” distribution of cities with high environmental quality in terms of “production, living and ecology” from 2010 to 2020 (Figure 3). The four temporal maps show that the cities with high production, living and ecological quality are particularly concentrated in China’s coastal areas, the Pearl River Delta and inland provincial capitals. First, the Northeast was examined. The Hadazhi urban agglomeration, which includes the cities of Changchun and Harbin. The south-central Liaoning urban agglomeration, including the cities of Dalian and Shenyang. Second, the eastern region. Beijing-Tianjin-Hebei City Cluster, including the cities of Beijing, Tianjin, Shijiazhuang, Tangshan and Baoding. The Yangtze River Delta city cluster, including the cities of Shanghai, Suzhou, Nanjing, Hangzhou, Ningbo, Wuxi, Nantong, Wenzhou, Changzhou, Jiaxing, Shaoxing, Xuzhou, Taizhou, Jinhua, Yancheng, Yangzhou, Taizhou and Huzhou. Pearl River Delta city cluster, including Guangzhou, Shenzhen, Dongguan, Foshan, Huizhou, Zhuhai, Shantou, Jieyang, Zhongshan, Jiangmen and other cities. The city cluster on the west coast of the Strait, including the cities of Xiamen, Fuzhou, Quanzhou and Zhangzhou. Shandong Peninsula City Cluster, including cities such as Qingdao and Jinan. Third, the central region. Wuhan City Cluster has only 1 city in Wuhan, Central Plains City Cluster has only 1 city in Zhengzhou, and Changzhutan City Cluster has only 1 city in Changsha. Fourth, the western region. The Chengdu-Chongqing City Cluster, which includes two cities, Chongqing and Chengdu. There are also provincial capital cities such as Kunming, Guiyang, Urumqi, Hohhot and Xining with high overall PLE scores.

Analysis of the spatial distribution characteristics of cities with high production, living and ecological environmental quality. Firstly, the spatial pattern shows a significant spatial gradient of PLE scores from east to central to west, with the PLE scores decreasing as we move towards the western regions. Secondly, although the PLE composite score of the eastern coastal region is much higher than that of the central and western regions, the spatial differences are significant. It can be divided into two types: multinuclear and binuclear. The former is typified by the Yangtze River Delta city cluster and the Pearl River Delta city cluster, both of which have spatially contiguous cities with high PLE scores, while the latter is typified by the Shandong Peninsula city cluster, who’s high PLE scores are characterized by the “Jinan-Qingdao” twin core. Thirdly, most of the cities in the western region are spatially distributed in a double-core or single-core pattern. The Chengdu-Chongqing urban agglomeration is typical, while the Guanzhong urban agglomeration and the Beibu Gulf urban agglomeration are typical of the single-core spatial distribution. The spatial distribution of the above cities is basically consistent with the strategic spatial layout of the large urban agglomerations approved by the state, indicating that there is a great consistency between the level of urbanization and the quality of the urban “production, living and ecological” environment. The higher the level of urbanization, the higher the overall score of “production, living and ecological” environmental quality of the city.

### 3.5. A Logarithmic Relationship between the Quality of the Urban “Production, Living and Ecological” Environment and the Size of the Built-Up Area

#### 3.5.1. Analysis of Total Sample Data

**Figure 4:**
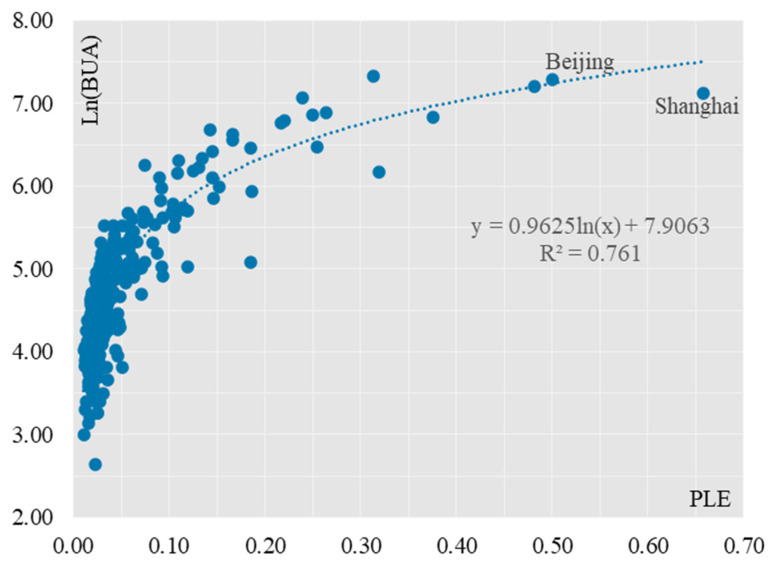
Scatterplot of the two variables PLE and BUA (analysis of 285 cities).

The built-up area is the most intuitive representation of the urbanization of a physical territory, reflecting the development process of the urbanization of the geographical landscape. The rapid urbanization and industrialization process has resulted in a rapid expansion of urban built-up land area and a significant increase in the size and number of towns and cities. Therefore, the built-up area is used here to analyses the factors related to the quality of the urban “production, living and ecological” environment. The simulations between the two variables of urban “production, living and ecological” environmental quality and built-up area show that when the independent variable x is PLE, the logarithmic model has the highest fit with an R^2^ of 0.761 (Figure 4), while the exponential model, linear model, binomial model and power model have a relatively lower fit than the logarithmic model, with an R^2^ of 0.4 for each of them. with R^2^ of 0.446, 0.553, 0.710 and 0.756 respectively. When the independent variable x was BUA, the exponential model had the highest fit with an R^2^ of 0.799, while the logarithmic, linear, binomial and power models had relatively lower fits than the exponential model, with R^2^ of 0.467, 0.553, 0.782 and 0.736 respectively (Table 7).

The best fit between PLE and built-up area is a logarithmic function when the independent variable is the city’s “production, living and ecological” environmental quality score, the built-up area increases rapidly with the city’s “production, living and ecological” environmental quality score. As the relationship is logarithmic, when the PLE score rises to a certain level, the growth in built-up area slows down, meaning that the growth in built-up area is not infinite, but has a certain limit. In social practice, the Chinese government has implemented a strict arable land protection system, strictly adhered to the red line of 1.8 billion mu of arable land, controlled disorderly urban expansion, vigorously improved the intensification and efficiency of urban land use, and strengthened the promotion of new urbanization. When the independent variable is the built-up area, the best fit between PLE and built-up area is an exponential function, as the built-up area increases rapidly, the overall score of “production, living and ecological” environmental quality of the city improves significantly. Similarly, the new urbanization strategy places greater emphasis on the efficient and intensive use of land, and protects permanent ecological forests and ten thousand mu of farmland from uncontrolled development, so that the built-up area cannot grow indefinitely. This is also illustrated by the counterfactual method, where the economic and social development of a city is ideally in harmony with its population and resources, and where the quality of the city’s “production, living and ecological” environment is developed in a synergistic manner to make it more viable. If a city is developed in a disorderly manner, with a large amount of real estate, industrial land, etc., but without the inflow of people, it will lead to idle resources and waste, which is an unsustainable growth pattern.

#### 3.5.2. Analysis by Population Size in Urban Areas

The relationship between the combined production, living and ecological environmental quality score and the built-up area of a city shows a logarithmic curve, but the relationship varies between the two for cities of different sizes. Based on this, the study further classifies 285 cities into two types: large cities and small and medium cities, and explores the correlation between the two in order to provide an intuitive basis for optimizing the environmental quality of urban “production, living and ecology”.

Firstly, the classification of the population size of urban areas. According to the Notice on Adjusting the Criteria for Classifying the Size of Cities issued by the State Council of China in 2014, cities with a resident population of 10 million or more in urban areas are classified as mega-cities, cities with a resident population of 5–10 million in urban areas are classified as very large cities, cities with a resident population of 3–5 million in urban areas are classified as Type I large cities, cities with a resident population of 1–3 million in urban areas are classified as Type II large cities, cities with a permanent urban population of 500,000–1 million are classified as medium-sized cities, and cities with a permanent urban population of less than 500,000 are classified as small cities. The 2020 China Census Sub-county Information published in October 2022 has a total of 685 cities in China, including 7 mega-cities (Shanghai, Beijing, Guangzhou, Shenzhen, Chongqing, Tianjin, Chengdu), 14 very large cities (Wuhan, Dongguan, Xi’an, Hangzhou, Foshan, Nanjing, Shenyang, Qingdao, Jinan, Changsha, Harbin, Zhengzhou, Kunming, Dalian), 14 Type I large cities (Nanning, Shijiazhuang, Xiamen, Taiyuan, Suzhou, Guiyang, Hefei, Urumqi, Ningbo, Wuxi, Fuzhou, Changchun, Nanchang, Changzhou), 70 Type II large cities and over 500 small and medium-sized cities. Since four of the 105 large cities are county-level cities, including Kunshan (1,143,300 people), Yiwu (1,184,200 people), Cixi (1,061,900 people) and Jinjiang (1,012,500 people), the environmental quality of urban “production, living and ecology” is only counted at the prefecture-level city level, so excluding these four cities, there are 101 large cities left.

**Figure 5:**
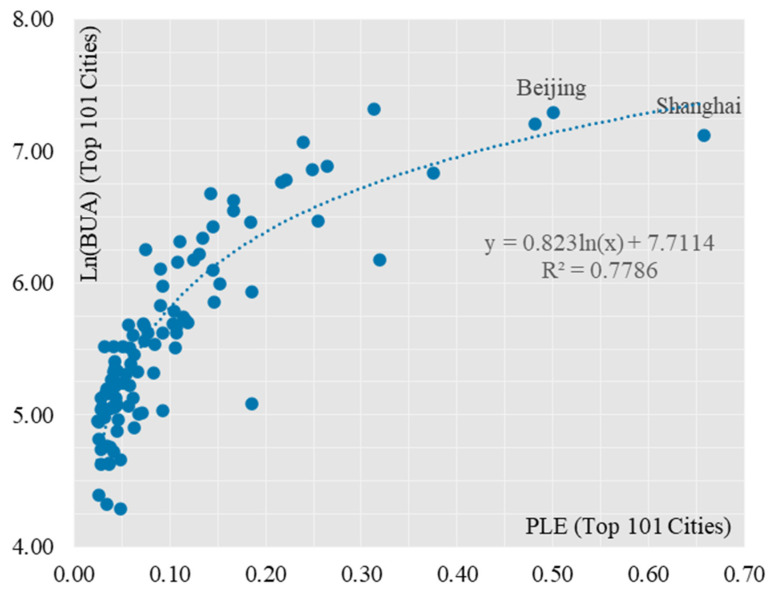
Scatterplot of two variables PLE and BUA (analysis of 101 large cities).

**Figure 6:**
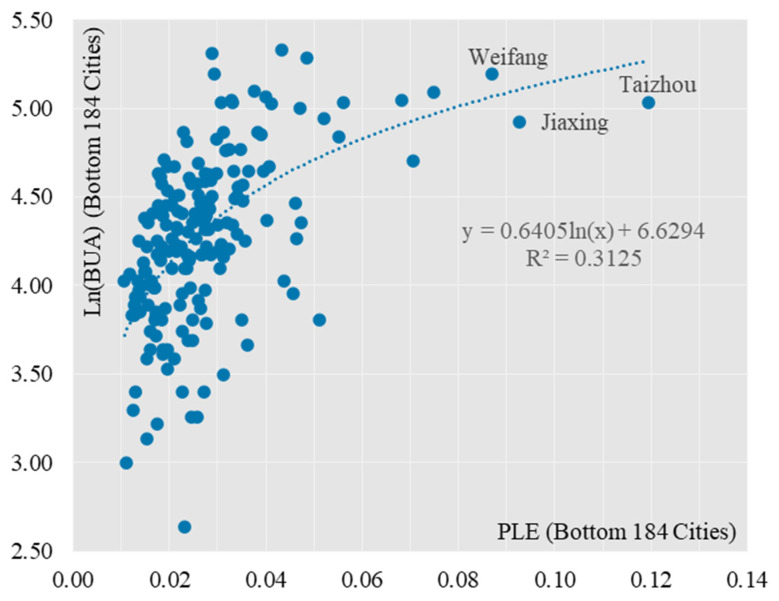
Scatterplot of two variables PLE and BUA (analysis of 184 small and medium-sized cities).

Secondly, the correlation analysis was carried out for the Top 101 and Bottom 184 cities respectively. 101 cities had a stronger logarithmic relationship between the combined PLE score and the built-up area (Figure 5), and the fit for 101 cities improved somewhat compared to 285 cities, with the R^2^ value increasing from 0.761 to 0.779. Most of the 101 cities are in the top 25% in terms of their combined PLE scores, a result that is highly consistent with the “clustered” spatial distribution of cities with the highest PLE scores. This result is highly consistent with the “cluster-like” spatial distribution of the cities with the highest PLE scores. The coefficient before the ln(x) function in the model decreases, from 0.9625 to 0.823, indicating that as the PLE score increases, the built-up area does not increase indefinitely, but has certain limits, as exemplified by the mega-cities of Beijing and Shanghai. For Bottom184 cities, the logarithmic relationship between the combined PLE score and built-up area for small and medium-sized cities has weakened (Figure 6), and the fit has dropped significantly, from 0.779 to 0.313, indicating that the logarithmic relationship between the combined PLE score and built-up area for small and medium-sized cities has dropped significantly. The log-curve relationship with built-up area is less significant, and the difference in PLE composite scores between the 184 small and medium-sized cities is much lower than that of the 101 large cities, with a coefficient of variation of 0.2899 for the former and 0.9447 for the latter. This result corresponds to the head effect of the combined PLE scores of production, living and ecology derived in the previous section.

## 4. Discussion and Conclusions

### 4.1. Discussion

In 1960, C.A. Doxiadis, founder of Ekistics, said at the fifth urban renewal working conference held in North Carolina in the United States: “When I was a six-year-old child and escaped from my home, I went to the square in front of our house, where pine trees were everywhere; I played with my friends without being disturbed. When I was 16, I saw the first group of workers coming to cut down 50% of the trees on the square to open up space for streets and cars. To open up space for streets and cars, I saw another group of people laying around the square when I was 26 Set up a street, erect a monument for a politician in the center of the square, and cut down the last trees. When I was 36 years old, I could only see the monument of a politician in the middle of the car as a transportation agent. When I was 46 years old, I saw the monument was taken away, a wide street cut into the square from the middle, and other places were paved into parking lots? No, worsening changes–everywhere. In the past 40 years, I have not seen that the gradual change of a city has created an improvement in conditions” [73]. When Doxiadis told the background of this paragraph, Greece was in a period of rapid urbanization. He pointed out that the urban conditions in which human beings live have deteriorated, the unprecedented growth of population, including the socialization of all political systems and social strata, and the emergence of machines in our lives are important reasons for these changes.

The same phenomenon occurs in Asia. When Mike Douglas examined the livability of such megacities as Bangkok, Ho Chi Minh, Jakarta and Manila, he pointed out that industrialization and urbanization are profoundly transforming the local social structure. In the past few decades, the urban population and economy of Southeast Asia have rapidly transferred to a few mega cities. Although these mega cities have made great achievements in economy, urban growth has brought challenges and pressures in housing, transportation system, water supply, drainage system, social services (education, health, natural environment) and other aspects [74]. These global cities (production centers) in the Asia Pacific region, on the one hand, attract foreign investment and provide a livable living environment for foreign investors; On the other hand, the gap between rich and poor has widened, and the competition between cities is fierce [75].

Since the founding of New China, China has made certain achievements in transforming old cities and building new towns. However, for a long time, “focusing on production and neglecting life”, focusing only on industrial output value and ignoring urban construction, coupled with the tight and uncertain policies on population control and residential migration, led to different degrees of housing difficulties, tight traffic, environmental degradation, increased unemployment and other problems in various cities [76]. In 2011, when China’s urbanization rate exceeded 50%, large urban agglomerations became the core areas of the national economy. However, Chinese cities were characterized by large scale, fast speed and significant semi urbanization. The above urban problems were primarily left over from history. In the era of planned economy, China’s “focus on production, light of life” has brought about a serious lag in urban construction, resulting in housing shortage, environmental pollution, traffic congestion and other urban problems. Since the reform and opening up period, with the improvement of China’s opening up level, cities have developed rapidly. However, in just a few decades, China has caught up with the hundreds of years of development in developed countries. Urbanization, suburbanization and re urbanization have gone hand in hand. Due to the synchronous appearance of “time compression” effect and social transformation, system and other reasons, urban problems originally belonging to different development stages led to a concentrated outbreak of various urban problems [77], which was reflected in the unbalanced development of urban “production, life and ecological” environmental quality.

The deep reason for the change of urban “production, living and ecological” environmental quality is the interweaving and promotion of industrialization and urbanization. Industrialization is also linked with globalization, which together cause profound changes in the “production, living and ecological” environmental quality of Asian cities. Due to multiple reasons such as economic transformation and development, political system reform, opening to the outside world, and world industrial specialization, the environmental quality of “production, life, and ecology” in Chinese cities has been accelerated. At the technical level, due to different starting points, there are also large differences in the urban “production, living, ecological” environmental evaluation index system. The high value areas of the ecological space quality index are mainly concentrated in the northeast and southeast coastal areas. Our research also shows that the characteristics of spatial gradient in the eastern, central and western regions are significant. The environmental quality of “production, life and ecology” in the eastern region is the highest, followed by the central region, and again in the western region. However, we take cities as the research object, which is more detailed in spatial scale compared with Li’s research [43].

From the theoretical perspective of “elements structure function”, Liu (2017) used the national land use data to evaluate the utilization of “production, living and ecology” space in 1990 and 2010, and concluded that the production space is mainly distributed in the main urban agglomerations in the southeast of the Hu Huanyong Line, the living space is mainly concentrated in the major cities and urban agglomerations in China, and the ecological environment is mainly distributed in the northwest of the Hu Huanyong Line [46]. Our research is similar to that of Liu (2017) in that urban “production” environmental quality and “life” environmental quality are concentrated in major cities and urban agglomerations. There are two different places. On the one hand, we have used the latest data for analysis and will update our research to 2020; On the other hand, we have compared the comprehensive score of urban “production, living and ecological” environmental quality with the city size, revealing the important influence of urbanization factors on the urban “production, living and ecological” environmental quality, and pointing out the logarithmic curve relationship between the built-up area and the urban “production, living and ecological” environmental quality, which is more obvious in large cities and megacities.

Of course, our research is also insufficient. Although the research reveals the temporal and spatial pattern of urban “production, life and ecology” environmental quality from the urban level, there is still a lack of dynamic mechanism analysis of urban “production, life and ecology” environmental quality change. In the discussion part, it is mentioned that industrialization and globalization have the power of changing each other and have an impact on the urban “production, life and ecological” environmental quality. However, this impact should be analyzed in combination with the national conditions and the background of the times. Through field research, micro data can be obtained to reveal the micro mechanism of urban “production, life and ecological” environmental quality changes.

### 4.2. Conclusions

In this paper, we use the entropy method, the Theil index and correlation analysis, the study analyzed the spatial and temporal differences in “production, living and ecological” environmental quality of 285 cities in China from 2010 to 2020, and reached the following main conclusions.

(1) In terms of overall differences, the overall differences in the “production, living and ecological” environmental quality indices of the 285 cities in the study period underwent a process of “narrowing–widening–narrowing”. The differences within the four major zones of the country are much higher than those between the four major zones, and the differences within the zones show an increasing trend year by year. The intra-belt contribution rate is, in descending order, Eastern Region > Western Region > Central Region > Northeast Region. Intra-provincial variation in the eastern region is the main driver, while the contribution of intra-difference in the northeastern region is the smallest, and the contribution of intra-difference in the central and western regions is second only to that of the eastern region.

(2) In terms of time series differences, the overall scores of “production, living and ecological” environmental quality in the 285 cities in the study period show a decreasing trend, and the contribution of the PLE subsystem scores are, in descending order, production environmental quality > living environmental quality > ecological environmental quality, reflecting that the overall level of “production, living and ecological” environmental quality in the country is not satisfactory. “The unbalanced development between the production environment, living environment and ecological environment is still a prominent problem. Although the government is determined to continue to tackle the problem of ecological pollution during the study period and has achieved certain results, the current economic structure of China is undergoing a deep transformation and the industrial structure has not yet been renewed, and the transformation and upgrading pains of the energy and industrial structures are the underlying causes of the imbalance in the quality of the urban “production, living and ecological” environment.

(3) In terms of overall ranking, the headline effect of the PLE score is significant during the study period, with the top 10 cities being all mega-cities and megacities in China, with Shanghai, Beijing, Guangzhou and Shenzhen remaining in the top 4. The bottom 10 cities in the composite score are all small and medium-sized cities with significant regionalization characteristics, and from 2010 to 2020 the bottom 10 cities in the PLE composite score gradually change from a westernized character to a westernized and northeasterlies character.

(4) In terms of spatial pattern, there is a significant spatial gradient in the east, central and western regions, with the four major regions having the highest to lowest overall scores in terms of “production, living and ecological” environmental quality: eastern region > central region > western region > northeastern region. Regions with high scores in the “production, living and ecological” environment can be classified into three types: multinuclear, binuclear and mononuclear. The cities with high environmental quality in terms of production, living and ecology are mainly concentrated in China’s coastal areas, the Pearl River Delta, inland provincial capitals and other urban agglomerations. Some cities in the western part of the country have seen their scores rise faster, while some cities in the northeast have seen their scores fall significantly.

(5) In terms of influencing factors, there is a logarithmic relationship between the combined PLE score and the area of the built-up area, while the area of the built-up area and the combined PLE score show an exponential curve. The logarithmic relationship between PLE and BUA is significantly influenced by the urban population control variable. The logarithmic relationship between PLE and BUA is weakened for small and medium-sized cities with an urban population of less than 1 million.

## Figures and Tables

**Table 1:** Indicator system for evaluating the environmental quality of “production, living and ecology” in cities.

Target Level	Secondary Indicators	Tertiary Indicators	Properties
Quality of Production Environment (QoP)	Industrial	Number of large-scale industrial enterprises	Positive
		Number of foreign-invested enterprises	Positive
	Wholesale and Retail	Retail sales of social consumer goods	Positive
		Wholesale and retail merchandise sales	Positive
	Post and Telecom	Postal revenue	Positive
		Telecommunication revenue	Positive
	Energy utilization	Industrial electricity consumption	Positive
		Liquefied petroleum gas supply	Positive
Quality of Living Environment (QoL)	Employment	Number of employees in employment	Positive
		Average wage	Positive
	Education	Number of students in school	Positive
		Number of tertiary institutions	Positive
	Medical	Number of hospital beds	Positive
		Number of doctors	Positive
	Social Security	Number of urban workers’ pension participants	Positive
		Number of urban workers’ medical insurance participants	Positive
Quality of Ecological Environment (QoE)	Greening	Area of green space	Positive
		Greening rate of built-up areas	Positive
		Park Green Space	Positive
	Industrial waste discharge	Industrial wastewater discharge	Negative
		Industrial Sulphur dioxide emissions	Negative
		Industrial fume and dust emissions	Negative
	Environmental Governance	Centralized treatment rate of sewage treatment plants	Positive
		Harmless disposal rate of domestic waste	Positive

**Table 2:** Theil Index from 2010 to 2020.

Indicator	2010	2011	2012	2013	2014	2015	2016	2017	2018	2019	2020
Theil Index	0.490	0.482	0.466	0.493	0.515	0.535	0.543	0.518	0.513	0.505	0.488

**Table 3:** Contribution of within-group and between-group variation in Theil’s index.

Theil	2010	2011	2012	2013	2014	2015	2016	2017	2018	2019	2020
T_db_	6.27%	6.46%	6.29%	5.99%	6.16%	5.68%	5.65%	5.38%	6.97%	5.64%	5.39%
T_d_	46.68%	46.25%	46.36%	46.36%	44.08%	44.76%	44.91%	44.31%	43.06%	46.73%	46.73%
T_z_	9.07%	10.02%	9.26%	9.39%	10.04%	10.00%	9.94%	10.22%	11.46%	9.76%	10.20%
T_x_	15.32%	15.74%	15.76%	15.82%	16.98%	16.84%	17.30%	17.13%	16.66%	17.02%	16.89%
T_WR_	77.34%	78.47%	77.67%	77.56%	77.26%	77.28%	77.80%	77.04%	78.15%	79.16%	79.20%
T_BR_	22.66%	21.53%	22.33%	22.44%	22.74%	22.72%	22.20%	22.96%	21.85%	20.84%	20.80%

**Table 4:** Scores for the three tiers of indicators (2010–2020).

Indices	2010	2011	2012	2013	2014	2015	2016	2017	2018	2019	2020
QoP	41.68%	43.36%	41.28%	41.51%	44.10%	44.84%	43.95%	43.28%	45.61%	43.93%	42.71%
QoL	37.80%	36.51%	36.26%	38.05%	39.24%	38.50%	39.75%	39.69%	37.31%	39.39%	38.92%
QoE	20.52%	20.13%	22.46%	20.44%	16.66%	16.66%	16.30%	17.03%	17.08%	16.68%	18.36%

**Table 5:** Top 10 cities and bottom 10 cities of PLE.

Order	2010	2011	2012	2013	2014	2015	2016	2017	2018	2019	2020
1	SH	SH	SH	SH	SH	SH	SH	SH	SH	SH	SH
2	BJ	BJ	BJ	BJ	BJ	BJ	BJ	BJ	BJ	BJ	BJ
3	GZ	GZ	GZ	GZ	GZ	GZ	GZ	GZ	GZ	GZ	GZ
4	SZ	SZ	SZ	SZ	SZ	SZ	SZ	SZ	SZ	SZ	SZ
5	TJ	CQ	CQ	CQ	CQ	CQ	CQ	CQ	SuZ	SuZ	SuZ
6	CQ	TJ	SuZ	SuZ	SuZ	SuZ	TJ	SuZ	CQ	CQ	CQ
7	SZ	SZ	TJ	TJ	TJ	TJ	SuZ	TJ	HZ	CD	CD
8	NJ	WH	HZ	NJ	HZ	HZ	HZ	HZ	TJ	HZ	HZ
9	HZ	NJ	WH	WH	NJ	DG	DG	DG	ZZ	DG	DG
10	WH	HZ	NJ	HZ	WH	NJ	NJ	CD	CD	TJ	TJ
276	BS	JYG	ZT	LN	YT	PE	BS	LY	WZ	LC	WZ
277	ZJJ	BS	BS	PE	PL	YC	WZ	SYS	LY	ZW	FCG
278	ZW	QTH	WZ	JC	JQ	BS	FCG	ZW	LN	TC	ZW
279	LC	ZW	PL	WZ	QTH	PL	LC	HG	TC	JC	JC
280	PL	ZY	QTH	QTH	BS	LC	LJ	PE	FCG	SYS	TC
281	QTH	PL	JC	BS	ZY	FCG	GY	TC	SYS	PE	PE
282	CZ	LN	FCG	FCG	YC	JC	JC	FCG	HG	FCG	LY
283	ZY	WZ	ZY	ZY	JC	QTH	HH	HH	QTH	LY	SYS
284	LN	JC	LJ	LJ	LC	LN	QTH	QTH	HH	HH	HH
285	FCG	FCG	GY	PL	LN	JYG	LN	JC	JC	HG	HG

Note: SH, BJ, GZ, SZ, TJ, CQ, SuZ, WH, DG, CD, ZZ refer, respectively, to Shanghai, Beijing, Guangzhou, Shenzhen, Tianjin, Chongqing, Suzhou, Wuhan, Dongguan, Chengdu, Zhengzhou; BS, JYG, ZT, LN, YT, PE, LY, WZ, LC, ZJJ, QTH, HH, PL, ZY, FCG, YC, GY, LN, JC, HG, TC, CZ refer, respectively, to Baoshan, Jiayuguan, Zhaotong, Longnan, Yingtan, Pu’er, Liaoyuan, Wuzhong, Lincang, Zhangjiaji, Qitaihe, Heihe, Pingliang, Zhangye, Fangchenggang, Yichun, Guyuan, Longnan, Jinchang, Hegang, Tongchuan, Chongzuo.

**Table 6:** Overall score of PLE.

Year	Overall Score	Northeast Region	Eastern Region	Central Region	Western Region
2010	Top 25%	6	47	8	10
	Middle 50%	18	37	62	25
	Bottom 25%	10	2	10	50
2013	Top 25%	5	48	9	9
	Middle 50%	16	36	57	33
	Bottom 25%	13	2	14	43
2016	Top 25%	4	48	8	11
	Middle 50%	17	38	59	28
	Bottom 25%	13	0	13	46
2020	Top 25%	4	45	11	11
	Middle 50%	10	38	57	37
	Bottom 25%	20	3	12	37

**Table 7:** Fit between two variables BUA and PLE.

Type	Model (*x*: PLE)	R^2^	Model (*x*: BUA)	R^2^
Logarithmic Curve	y = 0.9625 ln(*x*) + 7.9063	0.761	y = 0.2906 ln(*x*) − 0.3926	0.467
Exponential Curve	y = 4.2698 e^1.6061*x*^	0.446	y = 0.0009 e^0.7907*x*^	0.799
Linear	y = 8.5063*x* + 4.2682	0.553	y = 0.065*x* − 0.2529	0.553
Binomial Curve	y = −22.728*x*^2^ + 18.364*x* + 3.9201	0.710	y = 0.0349*x*^2^ − 0.2881*x* + 0.6108	0.782
Multiplicative Power	y = 8.7353*x*^0.1905^	0.756	y = 0.0001*x*^3.7054^	0.736

## Data Availability

No new data were created or analyzed in this study. Data sharing is not applicable to this article.
